# Whole-Genome Sequence of *Mycoplasma bovis* Strain Ningxia-1

**DOI:** 10.1128/genomeA.01367-17

**Published:** 2018-01-25

**Authors:** Peng Sun, Haifeng Luo, Xin Zhang, Jingyi Xu, Yanan Guo, Shenghu He

**Affiliations:** a School of Agriculture, Ningxia University, Yinchuan, China; b Virginia-Maryland Regional College of Veterinary Medicine, University of Maryland–College Park, College Park, Maryland, USA

## Abstract

A genome sequence of the *Mycoplasma bovis* Ningxia-1 strain was tested by Pacific Biosciences (PacBio) single-molecule real-time (SMRT) sequencing technology. The strain was isolated from a lesioned calf lung in 2013 in Pengyang, Ningxia, China. The single circular chromosome of 1,033,629 bp shows differences between complete *Mycoplasma bovis* genome in insertion-like sequences (ISs), integrative conjugative elements (ICEs), lipoproteins (LPs), variable surface lipoproteins (VSPs), pathogenicity islands (PAIs), etc.

## GENOME ANNOUNCEMENT

*Mycoplasma bovis* is the main cause of bovine respiratory disease syndrome. At present, the pathogen is prevalent worldwide, which causes huge economic losses to national cattle industries (1). In 2008, *M. bovis* was isolated from beef cattle in Hubei, China (2). Other places in China, such as Xinjiang, Ningxia, Chongqing, Guizhou, and Qingdao, later reported *M. bovis* isolated from beef cattle and dairy cows (3).

The whole-genome sequence of the *M. bovis* Ningxia-1 strain was isolated in 2013 from the lesioned lung of a beef calf, which was tested using single-molecule real-time (SMRT) technology (4), resulting in approximately 1,262-fold final sequence coverage. The genome of *M. bovis* Ningxia-1 contains a single circular chromosome of 1,033,629 bp, with a GC content of 29.33%. A total of 754 open reading frames (ORFs) were identified, totaling 845,928 bp (maximum length, 9,981 bp; minimum length, 114 bp), and occupied 81.84% of the whole genome, with an average length of 1121 bp and a mean GC content of 29.77%. A total of 577 coding sequence (CDS) genes could be classified into clusters of orthologous groups (COG) families, which have 19 functional categories. Seventy-four pseudogenes were predicted by GeneMarkS+ (5). The genome encodes 6 rRNA and 34 tRNA genes, representing all 20 amino acids.

We found 60 insertion-like sequence (IS) elements that comprised three distinct categories (https://www-is.biotoul.fr). A cluster of 8 variable surface protein (VSP)-related ORFs were found in the genome. Sixty-six lipoproteins (LPs) revealed signatures denoting distinct mutation-based mechanisms of phase variation (6). Of three predicted pathogenicity islands (PAIs) (7) with a lower GC content (8), PAI-1 (463,228 to 485,927) encoded one single-stranded DNA-binding protein and one conjugal transfer protein, TraE, and contained a transposase at the 5′ end. PAI-2 (541731 to 556809) presented an IS*4* and IS*30* family transposase at the 3′ end and contained an IS*1634* family transposase gene. A total of two nucleotidyl transferase AbiEii/AbiGii toxin family proteins and abortive phage infection proteins in PAI-3 (968,698 to 977,477) were without an IS element but still denoted a pathogenicity island. These characteristics may contribute to the emergence of bacterial pathogens with new virulence properties (9).

### Accession number(s).

This whole-genome sequence assembly has been deposited at GenBank under the accession no. CP023663.
